# Supplementary figures and images for: RCFGL: Rapid Condition adaptive Fused Graphical Lasso and application to modeling brain region co-expression networks
Source: PLoS Comput Biol. 2023 Jan 6;19(1):e1010758. doi: 10.1371/journal.pcbi.1010758 (PMC9821764; doi:10.1371/journal.pcbi.1010758)

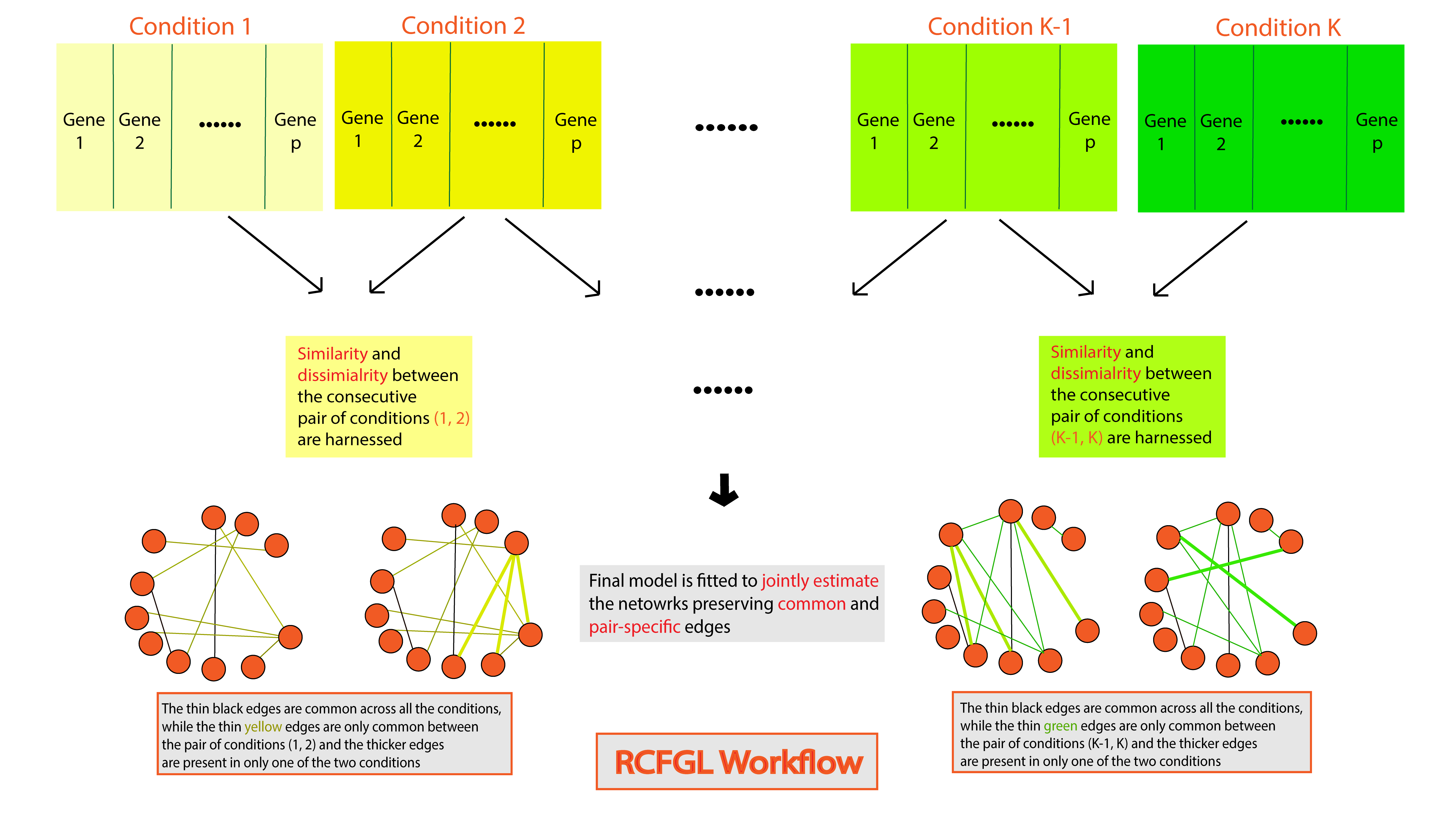

Supplement: S1 Fig — Expression data of multiple (p) genes are available in multiple (K) conditions at the start. In the next step, pair-specific patterns of similarity and dissimilarity between consecutive pairs of conditions are explored. In the final step the full model is fitted to jointly estimate all the networks using the proposed model. (TIFF) [file pcbi.1010758.s001.tiff]
