# Supplementary material for: RCFGL: Rapid Condition adaptive Fused Graphical Lasso and application to modeling brain region co-expression networks
Source: PLoS Comput Biol. 2023 Jan 6;19(1):e1010758. doi: 10.1371/journal.pcbi.1010758 (PMC9821764; doi:10.1371/journal.pcbi.1010758)
Supplement: S1 Text — We provide the proof of the theorem for detecting block-diagonal structure in the precision matrices and derive the connections between the penalty terms used in different methods. We also list the quality control steps used in the real data pruning. (PDF) [file pcbi.1010758.s003.pdf]

S1 Text

Souvik Seal, Qunhua Li, Elle Butler Basner, Laura Saba, Katerina Kechris

December 5, 2022

1 Theorem for detecting block-diagonal structure

Theorem 1. *Denote the set of p genes by C. Suppose, p_1 genes belong to a set C_1 and p_2 belong to a set C_2. And, $C_1 \cap C_2 = \phi$ and $C_1 \cup C_2 = C$. For the genes in C_1 to be completely disconnected from those in C_2 in each of the resulting network estimates using RCFGL, it is sufficient that $|n_k \mathbf{S}_{ij}^{(k)}| < \lambda_1$ for $k = 1, 2, \cdots, K$ and $i \in C_1, j \in C_2$.*

Proof. Suppose there are $K = 2$ classes. By the Karush-Kuhn-Tucker (KKT; [1]) conditions, a necessary and sufficient set of conditions for $\{\Theta\}$ to be the solution to the RCFGL problem is that,

$$\begin{aligned} 0 &= n_1(\mathbf{\Theta}^1)^{-1} - n_1\mathbf{S}^{(1)} - \lambda_1\Gamma_1 - \lambda_2\mathbf{W} \odot \Upsilon \\ 0 &= n_2(\mathbf{\Theta}^2)^{-1} - n_2\mathbf{S}^{(2)} - \lambda_1\Gamma_2 + \lambda_2\mathbf{W} \odot \Upsilon \end{aligned} \tag{1}$$

where $\Gamma_{1,ij}, \Gamma_{2,ij}$ are the sub-gradients of $|\theta_{ij}^{(1)}|$, $|\theta_{ij}^{(2)}|$ w.r.t $\theta_{ij}^{(1)}, \theta_{ij}^{(2)}$ respectively, and Υ_{ij} is the sub-gradient of $|\theta_{ij}^{(1)} - \theta_{ij}^{(2)}|$ w.r.t $\theta_{ij}^{(1)}$. Consider the matrices,

$$\mathbf{\Theta}^{(1)} = \begin{bmatrix} \mathbf{\Theta}_1^{(1)} & \mathbf{0} \\ \mathbf{0} & \mathbf{\Theta}_2^{(1)} \end{bmatrix}, \mathbf{\Theta}^{(2)} = \begin{bmatrix} \mathbf{\Theta}_1^{(2)} & \mathbf{0} \\ \mathbf{0} & \mathbf{\Theta}_2^{(2)} \end{bmatrix} \tag{2}$$

where $\mathbf{\Theta}_1^{(1)}, \mathbf{\Theta}_1^{(2)}$ solve the RCFGL problem on the genes in C_1, and where $\mathbf{\Theta}_2^{(1)}, \mathbf{\Theta}_2^{(2)}$ solve the RCFGL problem on the genes in C_2. Inspecting Eq. 2, $\mathbf{\Theta}^{(1)}, \mathbf{\Theta}^{(2)}$ solves the entire RCFGL problem if and only if for all $i \in C_1, j \in C_2$, there exist $\Gamma_{1,ij}, \Gamma_{2,ij}, \Upsilon_{ij} \in [-1, 1]$ such that,

$$\begin{aligned} &- n_1\mathbf{S}_{ij}^{(1)} - \lambda_1\Gamma_{1,ij} - \lambda_2 w_{ij}^{12} \cdot \Upsilon_{ij} = 0 \\ &- n_2\mathbf{S}_{ij}^{(2)} - \lambda_1\Gamma_{2,ij} + \lambda_2 w_{ij}^{12} \cdot \Upsilon_{ij} = 0 \end{aligned} \tag{3}$$

So, we need to find out the solutions of $\Gamma_{1,ij}, \Gamma_{2,ij}, \Upsilon_{ij}$ that would satisfy Eq. 3. If we set $\Upsilon_{ij} = 0$, Eq. 3 will reduce to,

$$-n_1\mathbf{S}_{ij}^{(1)} - \lambda_1\Gamma_{1,ij} = 0 \quad \text{and} \quad - n_2\mathbf{S}_{ij}^{(2)} - \lambda_1\Gamma_{2,ij} = 0$$

giving the solutions to $\Gamma_{1,ij}, \Gamma_{2,ij}$ as $\Gamma_{1,ij} = -\frac{n_1}{\lambda_1}\mathbf{S}_{ij}^{(1)}$ and $\Gamma_{2,ij} = -\frac{n_2}{\lambda_1}\mathbf{S}_{ij}^{(2)}$. If the inequalities: $|n_1\mathbf{S}_{ij}^{(1)}| < \lambda_1$, $|n_2\mathbf{S}_{ij}^{(2)}| < \lambda_1$ hold, it is obvious that $\Gamma_{1,ij}, \Gamma_{2,ij} \in [-1, 1]$. Thus, $\mathbf{\Theta}^{(1)}$, $\mathbf{\Theta}^{(2)}$ from Eq. 1 will also be the solution of the entire RCFGL under those two particular inequalities. The proof is similar for $K > 2$ classes and is omitted here. □

2 Connection between FGL and FMGL penalties

$$\mathrm{P}^{\mathrm{FGL}}(\mathbf{\Theta}, \lambda_1, \lambda_2) = \lambda_1 \sum_{i \neq j} \sum_{k=1}^{K} |\mathbf{\Theta}_{ij}^{(k)}| + \lambda_2 \sum_{i \neq j} \sum_{k<k'}^{K} |\mathbf{\Theta}_{ij}^{(k)} - \mathbf{\Theta}_{ij}^{(k')}|;$$

$$\mathrm{P}^{\mathrm{FMGL}}(\mathbf{\Theta}, \lambda_1, \lambda_2) = \lambda_1 \sum_{i \neq j} \sum_{k=1}^{K} |\mathbf{\Theta}_{ij}^{(k)}| + \lambda_2 \sum_{i \neq j} \sum_{k=1}^{K-1} |\mathbf{\Theta}_{ij}^{(k)} - \mathbf{\Theta}_{ij}^{(k+1)}|;$$

For $K = 3$, we investigate the second term of $\mathrm{P}^{\mathrm{FGL}}(\boldsymbol{\Theta})$ focusing on the ij-th summand at a time,

$$\begin{aligned}\sum_{k<k'}^{3} |\boldsymbol{\Theta}_{ij}^{(k)} - \boldsymbol{\Theta}_{ij}^{(k')}| &= |\boldsymbol{\Theta}_{ij}^{(1)} - \boldsymbol{\Theta}_{ij}^{(2)}| + |\boldsymbol{\Theta}_{ij}^{(2)} - \boldsymbol{\Theta}_{ij}^{(3)}| + |\boldsymbol{\Theta}_{ij}^{(1)} - \boldsymbol{\Theta}_{ij}^{(3)}| \\ &\leq |\boldsymbol{\Theta}_{ij}^{(1)} - \boldsymbol{\Theta}_{ij}^{(2)}| + |\boldsymbol{\Theta}_{ij}^{(2)} - \boldsymbol{\Theta}_{ij}^{(3)}| + |\boldsymbol{\Theta}_{ij}^{(1)} - \boldsymbol{\Theta}_{ij}^{(2)}| + |\boldsymbol{\Theta}_{ij}^{(2)} - \boldsymbol{\Theta}_{ij}^{(3)}| \\ &= 2|\boldsymbol{\Theta}_{ij}^{(1)} - \boldsymbol{\Theta}_{ij}^{(2)}| + 2|\boldsymbol{\Theta}_{ij}^{(2)} - \boldsymbol{\Theta}_{ij}^{(3)}| \\ &= 2\sum_{k=1}^{2} |\boldsymbol{\Theta}_{ij}^{(k)} - \boldsymbol{\Theta}_{ij}^{(k+1)}|\end{aligned}$$

We have used the triangle inequality: $|\boldsymbol{\Theta}_{ij}^{(1)} - \boldsymbol{\Theta}_{ij}^{(3)}| \leq |\boldsymbol{\Theta}_{ij}^{(1)} - \boldsymbol{\Theta}_{ij}^{(2)}| + |\boldsymbol{\Theta}_{ij}^{(2)} - \boldsymbol{\Theta}_{ij}^{(3)}|$. Summing all ij terms we get,

$$\sum_{i\neq j}\sum_{k<k'}^{3} |\boldsymbol{\Theta}_{ij}^{(k)} - \boldsymbol{\Theta}_{ij}^{(k')}| \leq 2\sum_{i\neq j}\sum_{k=1}^{2} |\boldsymbol{\Theta}_{ij}^{(k)} - \boldsymbol{\Theta}_{ij}^{(k+1)}|.$$

And, finally we establish the connection between $\mathrm{P}^{\mathrm{FGL}}$ and $\mathrm{P}^{\mathrm{FMGL}}$ as,

$$\begin{aligned}\mathrm{P}^{\mathrm{FGL}}(\boldsymbol{\Theta}, \lambda_1, \lambda_2) &= \lambda_1 \sum_{i\neq j}\sum_{k=1}^{3} |\boldsymbol{\Theta}_{ij}^{(k)}| + \lambda_2 \sum_{i\neq j}\sum_{k<k'}^{3} |\boldsymbol{\Theta}_{ij}^{(k)} - \boldsymbol{\Theta}_{ij}^{(k')}| \\ &\leq \lambda_1 \sum_{i\neq j}\sum_{k=1}^{3} |\boldsymbol{\Theta}_{ij}^{(k)}| + 2\lambda_2 \sum_{i\neq j}\sum_{k=1}^{2} |\boldsymbol{\Theta}_{ij}^{(k)} - \boldsymbol{\Theta}_{ij}^{(k+1)}| \\ &= \mathrm{P}^{\mathrm{FMGL}}(\boldsymbol{\Theta}, \lambda_1, 2\lambda_2).\end{aligned}$$

For $K > 3$, using similar idea we can find a crude bound,

$$\mathrm{P}^{\mathrm{FGL}}(\boldsymbol{\Theta}, \lambda_1, \lambda_2) \leq \mathrm{P}^{\mathrm{FMGL}}(\boldsymbol{\Theta}, \lambda_1, \left\lfloor \frac{K^2}{4} \right\rfloor \lambda_2).$$

3 Connection between CFGL and RCFGL penalties

$$\begin{aligned}\mathrm{P}^{\mathrm{CFGL}}(\boldsymbol{\Theta}, \lambda_1, \lambda_2, \mathbf{W}) &= \lambda_1 \sum_{i\neq j}\sum_{k=1}^{K} |\boldsymbol{\Theta}_{ij}^{(k)}| + \lambda_2 \sum_{i\neq j}\sum_{k<k'}^{K} \mathbf{w}_{ij}^{(kk')} |\boldsymbol{\Theta}_{ij}^{(k)} - \boldsymbol{\Theta}_{ij}^{(k')}|; \\ \mathrm{P}^{\mathrm{RCFGL}}(\boldsymbol{\Theta}, \lambda_1, \lambda_2, \mathbf{W}) &= \lambda_1 \sum_{i\neq j}\sum_{k=1}^{K} |\boldsymbol{\Theta}_{ij}^{(k)}| + \lambda_2 \sum_{i\neq j}\sum_{k=1}^{K-1} \mathbf{w}_{ij}^{(kk+1)} |\boldsymbol{\Theta}_{ij}^{(k)} - \boldsymbol{\Theta}_{ij}^{(k+1)}|;\end{aligned}$$

For $K = 3$, we investigate the second term of $\mathrm{P}^{\mathrm{FGL}}(\boldsymbol{\Theta})$ focusing on the ij-th summand at a time,

$$\begin{aligned}\sum_{k<k'}^{3} \mathbf{w}_{ij}^{(kk')} |\boldsymbol{\Theta}_{ij}^{(k)} - \boldsymbol{\Theta}_{ij}^{(k')}| &= \mathbf{w}_{ij}^{(12)} |\boldsymbol{\Theta}_{ij}^{(1)} - \boldsymbol{\Theta}_{ij}^{(2)}| + \mathbf{w}_{ij}^{(23)} |\boldsymbol{\Theta}_{ij}^{(2)} - \boldsymbol{\Theta}_{ij}^{(3)}| + \mathbf{w}_{ij}^{(13)} |\boldsymbol{\Theta}_{ij}^{(1)} - \boldsymbol{\Theta}_{ij}^{(3)}| \\ &\leq (\mathbf{w}_{ij}^{(12)} + \mathbf{w}_{ij}^{(13)}) |\boldsymbol{\Theta}_{ij}^{(1)} - \boldsymbol{\Theta}_{ij}^{(2)}| + (\mathbf{w}_{ij}^{(23)} + \mathbf{w}_{ij}^{(13)}) |\boldsymbol{\Theta}_{ij}^{(2)} - \boldsymbol{\Theta}_{ij}^{(3)}| \\ &= \mathbf{w}_{ij}^{*(12)} |\boldsymbol{\Theta}_{ij}^{(1)} - \boldsymbol{\Theta}_{ij}^{(2)}| + \mathbf{w}_{ij}^{*(23)} |\boldsymbol{\Theta}_{ij}^{(2)} - \boldsymbol{\Theta}_{ij}^{(3)}| \\ &= \sum_{k=1}^{2} \mathbf{w}_{ij}^{*(kk+1)} |\boldsymbol{\Theta}_{ij}^{(k)} - \boldsymbol{\Theta}_{ij}^{(k+1)}|.\end{aligned}$$

where, $\mathbf{w}_{ij}^{*(12)} = (\mathbf{w}_{ij}^{(12)} + \mathbf{w}_{ij}^{(13)})$ and $\mathbf{w}_{ij}^{*(23)} = (\mathbf{w}_{ij}^{(23)} + \mathbf{w}_{ij}^{(13)})$. Define two modified weight matrices as, $\mathbf{W}^{*(12)} = [[\mathbf{w}_{ij}^{*(12)}]]$ and $\mathbf{W}^{*(23)} = [[\mathbf{w}_{ij}^{*(23)}]]$. Denoting $\mathbf{W}^*$ to be the set of the modified weight matrices:

$\mathbf{W}^* = \{\mathbf{W}^{*(12)}, \mathbf{W}^{*(23)}\}$, we arrive at the following inequality,

$$\begin{aligned}\mathrm{P}^{\mathrm{CFGL}}(\boldsymbol{\Theta}, \lambda_1, \lambda_2, \mathbf{W}) &= \lambda_1 \sum_{i\neq j}\sum_{k=1}^{3} |\boldsymbol{\Theta}_{ij}^{(k)}| + \lambda_2 \sum_{i\neq j}\sum_{k<k'}^{3} \mathbf{w}_{ij}^{(kk')} |\boldsymbol{\Theta}_{ij}^{(k)} - \boldsymbol{\Theta}_{ij}^{(k')}| \\ &\leq \lambda_1 \sum_{i\neq j}\sum_{k=1}^{3} |\boldsymbol{\Theta}_{ij}^{(k)}| + \lambda_2 \sum_{i\neq j}\sum_{k=1}^{2} \mathbf{w}_{ij}^{*(kk+1)} |\boldsymbol{\Theta}_{ij}^{(k)} - \boldsymbol{\Theta}_{ij}^{(k+1)}| \\ &= \mathrm{P}^{\mathrm{RCFGL}}(\boldsymbol{\Theta}, \lambda_1, \lambda_2, \mathbf{W}^*).\end{aligned}$$

For $K > 3$, using similar idea we can show that,

$$\mathrm{P}^{\mathrm{FGL}}(\boldsymbol{\Theta}, \lambda_1, \lambda_2, \mathbf{W}) \leq \mathrm{P}^{\mathrm{RCFGL}}(\boldsymbol{\Theta}, \lambda_1, \lambda_2, \mathbf{W}^*); \quad \mathbf{W}^{*(kk+1)} = \sum_{r=1}^{k-1}\sum_{n=k+1}^{K} \mathbf{W}^{(rn)} + \sum_{n=k+1}^{K} \mathbf{W}^{(kn)}.$$

where, $\mathbf{W}^*$ is the set of the modified weight matrices, $\mathbf{W}^* = \{\mathbf{W}^{*(kk+1)} : k = 1, 2, \ldots, K-1\}$.

4 Quality control details of the real data

Raw sequencing data of this paper can be accessed through GSE173141. Initially, individual libraries were removed if their raw read count was less than 5 million, if more than 2% of reads were removed during trimming, if the average read length after trimming was less than 95 nucleotides, if less than 25% of trimmed reads aligned to the Ensembl transcriptome, if less than 50% of trimmed reads aligned to the reference genome, or if less than 50% of reads that aligned to the genome aligned uniquely. Next, libraries were eliminated from the three brain regions of interest because the genotype information derived from the RNA-Seq data did not match the genotype information from that rat derived from genotype-by-sequencing. Based on the same genotype comparison, two rat labels were swapped within the LHB data. Quality control measures are also documented in [2]. In the final analysis, we considered 64 rats whose IDs are listed below for the sake of reproducibility.

`00077E67B5, 00077E8336, 00077EA7E6, 000789FF7D, 00078A0041, 00078A0138, 00078A0189, 00078A01C0, 00078A0224, 00078A02CB, 00078A18A7, 00078A19A7, 00078A19C0, 00078A1F34, 00078A2315, 00078A2463, 00078A261F, 000789FFF0, 000789FFF9, 00078A0139, 00078A01DB, 00078A1707, 00078A1837, 00078A1A16, 000789FF6E, 000789FF94, 000789FFD3, 00078A0058, 00078A0085, 00078A0127, 00078A0166, 00078A01A6, 00078A01D8, 00078A01FE, 00078A021A, 00078A022D, 00078A0255, 00078A02DF, 00078A087B, 00078A09B1, 00078A0AEA, 00078A16D3, 00078A16DF, 00078A1731, 00078A1732, 00078A1772, 00078A179C, 00078A17F7, 00078A1807, 00078A181B, 00078A1863, 00078A186C, 00078A1875, 00078A18CF, 00078A18F7, 00078A192C, 00078A1937, 00078A1942, 00078A194B, 00078A1979, 00078A19B5, 00078A1A2B, 00078A22DF, 00078A22EB, 00078A2595`

References

[1] Stephen Boyd, Stephen P Boyd, and Lieven Vandenberghe. *Convex optimization.* Cambridge university press, 2004.

[2] Daniel Munro, Tengfei Wang, Apurva S Chitre, Oksana Polesskaya, Nava Ehsan, Jianjun Gao, Alexander Gusev, Leah C Solberg Woods, Laura M Saba, Hao Chen, et al. The regulatory landscape of multiple brain regions in outbred heterogeneous stock rats. *bioRxiv*, 2022.
