# Supplementary material for: RCFGL: Rapid Condition adaptive Fused Graphical Lasso and application to modeling brain region co-expression networks
Source: PLoS Comput Biol. 2023 Jan 6;19(1):e1010758. doi: 10.1371/journal.pcbi.1010758 (PMC9821764; doi:10.1371/journal.pcbi.1010758)
Supplement: Attachment — Submitted filename: Review_replies.pdf [file pcbi.1010758.s008.pdf]

Response to the comments for "RCFGL: Rapid Condition adaptive Fused Graphical Lasso and application to modeling brain region co-expression networks"

Souvik Seal[1], Qunhua Li[2], Elle Butler Basner[2], Laura Saba[3], and Katerina Kechris[1]

[1]Department of Biostatistics and Informatics, Colorado School of Public Health, University of Colorado Anschutz Medical Campus, Aurora, Colorado, USA

[2]Department of Statistics, Pennsylvania State University, Centre County, Pennsylvania, USA

[3]Skaggs School of Pharmacy and Pharmaceutical Sciences, University of Colorado Anschutz Medical Campus, Aurora, Colorado, USA

Reviewer 1

Major comments:

1. *"In my opinion, this article together with the wonderfully designed repository may be of interest to a wide range of biologists. I was fully satisfied with the detailed mathematical description of the method, but to broaden the scope of readers I would suggest adding didactic figures and schemes explaining the principle of the method. I would also expand a little for non-experienced readers on*

the concept and necessity of L1 and L2 regularizations."

Reply: We thank the reviewer for the helpful comment. We have now provided the following workflow figure as a supplementary S1 Fig, showcasing the basic idea behind the proposed method.

Figure S1: **Workflow of the proposed method.** Expression data of multiple (p) genes are available in multiple (K) conditions at the start. In the next step, pair-specific patterns of similarity and dissimilarity between consecutive pairs of conditions are explored. In the final step the full model is fitted to jointly estimate all the networks using the proposed model.

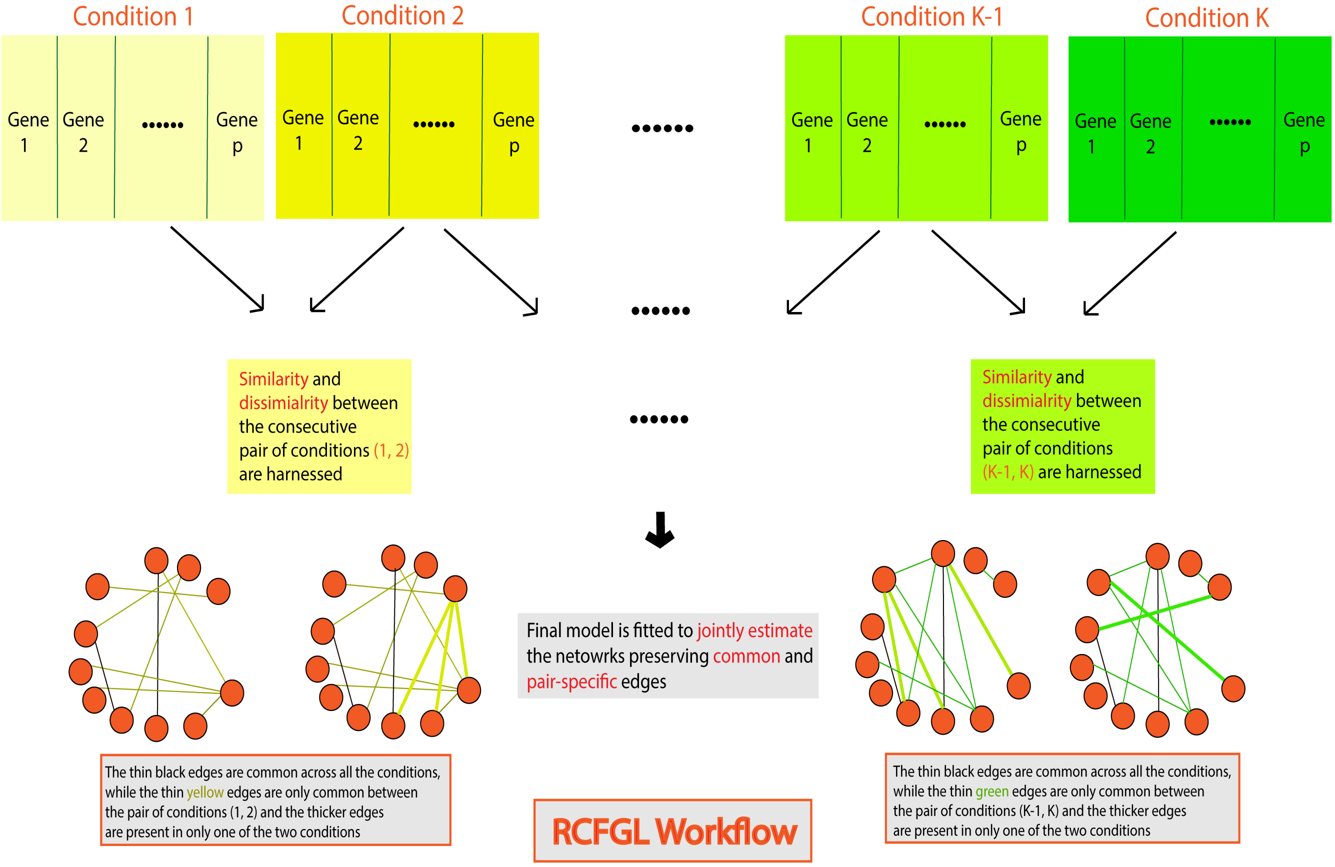

We have also addressed the reviewer's second comment by explaining the relevance of individual penalty terms in the Introduction section as:

"As the name suggests, FGL is an extension of the GL model in the context of multiple condi-

tions. It simultaneously estimates multiple precision matrices, corresponding to multiple conditions, by considering the sum of multiple GL likelihoods and further employs a standard lasso penalty [16] and a pairwise fused lasso penalty [17] across the conditions. The standard lasso penalty encourages sparsity in the network estimation and the pairwise fused lasso penalty ensures that the networks share some degree of similarity. "

2. *"Despite the publicly available and well-documented implementation of the method, I could not find any code for the figures in this article. Publishing the code is not only necessary as part of open science and journal policy, but will also greatly assist followers in discovering the potential of your method."*

Reply: We thank the reviewer for the comment. Following the reviewers' suggestions, we have now added fully commented codes used to generate and analyze the datasets of the paper in a folder named "Codes_used_in_paper" to our Github repository, https://github.com/sealx017/RCFGL. The data-generating codes are equipped with the "seeds" we originally used. The codes to generate the figures of the paper along with the extracted results (as rdata files) are also provided.

3. *"One of the main transcriptomic methods at the moment is single-cell sequencing. The data obtained by this method do not meet the assumptions of normality and often contain a significant amount of missing data. Despite the fact that such data were used in this article, I did not see any discussion of how sensitive the proposed method is to the assumption of normality and how to perform (and if at all) the imputation of missing measurements."*

Reply: We thank the reviewer for the comment. Our method is primarily suitable for estimating co-expression networks between genes from bulk RNA sequencing data, not single-cell RNA sequencing data, which may be zero inflated. Regarding normality of the gene expression-profiles, our method and the three other methods discussed in the paper are essentially based on the concept

of Gaussian graphical model (GGM) [3]. A typical remedy for dealing with violation of normality is to use transformations. In another project, we are investigating how a basic transformation-based approach, such as transforming non-normal data using a Box-Cox transformation and then using a GGM, compares to more sophisticated parametric models, such as combining multivariate Hurdle model and GGM [8].

4. *"Despite a good description of methods close to RCFGL, the authors left out a description and comparison with other methods of establishing differential co-regulatory networks, such as DiffCoEx, WGCNA, DICER, DINGO, etc."*

Reply: We thank the reviewer for pointing out a group of relevant methods for studying differential gene co-expression patterns between two conditions. Broadly, these methods follow two steps. First, the sample correlation of every two genes in each condition is used as a measure of their co-expression in that condition. This step is performed separately for each condition, and thus essentially implies that the network for every condition is getting constructed independently of the other conditions. Second, the sample correlation of those genes is compared between a pair of conditions to test if an edge is present in one or both conditions. As an example, DICER uses a t-test based probabilistic framework for this purpose. However, it has been shown that the methods like joint graphical model [6], FGL [4] and FMGL [18] which are based on estimating precision matrices using Gaussian graphical model (GGM) and incorporate the shared information across all the conditions, achieve significantly better statistical power. These sophisticated methods enable sparsity in the network estimation, preserve common network structure and generalize easily for more than two conditions. Out of the four methods the reviewer has mentioned, only DINGO achieves something similar. At the first step, it pools the data of every condition and estimates the global component i.e., the network structure present in every condition using a GGM framework.

In the next step, it estimates local condition-specific components using precision regression model. We believe that DINGO would achieve performance similar to FGL or FMGL. However, it can only handle a maximum of two conditions and thus, is less generalizable than the other two methods.

In this paper, our main goal was to address the limitation of FGL and FMGL which is the lack of the ability to take into account pair-specific patterns of similarities or dissimilarities between the conditions. We showed how our proposed method RCFGL which is conceptually similar to another existing method named CFGL, is more general and computationally tractable. Aligning with the reviewer's suggestion, we have now added the following paragraph in the introduction section of the manuscript:

"There are methods like DiffCoEx [15], DICER [1] and DINGO [7] which particularly aim to study such differential co-expression patterns between two conditions. Broadly, these methods compare the sample correlation of every pair of genes between two conditions. The problem with such approaches is two-fold: firstly, the sample correlation may not be an appropriate measure of co-expression in many datasets, especially with a large number of genes and a limited sample-size and secondly, with more than two conditions, the approaches compare the pairs of conditions independently and thereby failing to perform a joint comparison in the true sense."

5. *"One of the main claims of this article was the acceleration of existing methods. Unfortunately, these results were poorly expressed in the abstract, where I would add more concrete numbers on the increase in the speed of network construction with the proposed algorithm. I would also like to see a comparison of different algorithms, not only on simulated data but also on real data."*

Reply: We thank the reviewer for the comment. We have now adjusted the abstract appropriately highlighting the computational gains as below:

"Inferring gene co-expression networks is a useful process for understanding gene regulation

and pathway activity. The networks are usually undirected graphs where genes are represented as nodes and an edge represents a significant co-expression relationship. When expression data of multiple (p) genes in multiple (K) conditions (e.g., treatments, tissues, strains) are available, joint estimation of networks harnessing shared information across them can significantly increase the power of analysis. In addition, examining condition-specific patterns of co-expression can provide insights into the underlying cellular processes activated in a particular condition. Condition adaptive fused graphical lasso (CFGL) is an existing method that incorporates condition specificity in a fused graphical lasso (FGL) model for estimating multiple co-expression networks. However, with a computational complexity of $O(p^2 K \log K)$, the current implementation of CFGL is prohibitively slow even for a moderate number of genes and can only be used for a maximum of three conditions. In this paper, we propose a faster alternative of CFGL known as rapid condition adaptive fused graphical lasso (RCFGL). In RCFGL, we incorporate the condition specificity into another popular model for joint network estimation, known as fused multiple graphical lasso (FMGL). We use a more efficient algorithm in the iterative steps compared to CFGL, enabling faster computation with a complexity of $O(p^2 K)$ and making it easily generalizable for more than three conditions. We also present a novel screening rule to determine if the full network estimation problem can be broken down into estimation of smaller disjoint sub-networks, thereby reducing the complexity further. We demonstrate the computational advantage and superior performance of our method compared to two non-condition adaptive methods, FGL and FMGL, and one condition adaptive method, CFGL in both simulation study and real data analysis. We used RCFGL to jointly estimate the gene co-expression networks in different brain regions (conditions) using a cohort of heterogeneous stock rats. We also provide an accommodating *C* and *Python* based package that implements RCFGL."

In the real data analysis, we have provided the comparison of the run-times of the four methods for different gene-set sizes. We have also compared the edge-detection performance of the proposed

method with a non-condition adaptive method FMGL (referred to as RFGL in the manuscript). The other two methods could not be applied due to their excessive run-time requirement.

Minor comments:

1. *"After reading the paper, it remains unclear to me what happens if two genes in one group are linked positively and the other are linked negatively. Will such an edge be preserved as common for the two groups, or as different?"*

Reply: We thank the reviewer for raising an interesting point. In the scenario mentioned, our method will differentiate between the groups and not enforce any similarity between them for that edge. In other words, it will try to preserve the respective sign of the edge-weight in different groups.

2. *"Page 10, The legend to figure 1 could be more detailed."*

Reply: We thank the reviewer for the comment. We have added a few more lines to the description of Fig 1 as:

"Notice that in (S1), the first two conditions had the exactly same networks, whereas the network of the third condition was partially similar, sharing only the first three blocks. In (S2) and the following simulation scenarios, there was no such partial similarity considered and the conditions either shared the full network or they were entirely dissimilar."

3. *"Page 15, lines 444-446. Assessing the significance of observed differences would greatly strengthen the claim."*

Reply: We thank the reviewer for the great comment. We have now performed a pairwise t-test to determine the level of significance of the observed differences between the number of edges found to be unique to the pair (IL, PL) for three values of λ_2, 0.02, 0.03 and 0.04 as considered in Fig 8 of the main manuscript. We have added the p-values to the text as:

"We performed a pairwise t-test to determine statistical significance of this observation. For the three values of λ_2, 0.02, 0.03 and 0.04, the respective p-values were 0.01, 6e-12 and 2e-16, indicating increasing statistical significance of the difference between the numbers of IL-PL specific edges detected by RFGL and RCFGL."

4. *"Page 16, lines 465-467. Based on what data/reasoning was the number five chosen as the threshold for categorizing the gene as a hub?"*

Reply: We thank the reviewer for the comment. We chose five connections to define a node as a hub-gene just based on the average number of connections of the nodes with at least one connection, which in this case was 4.16. So, we defined the above-average nodes i.e, the ones with ≥ 5 connections to be hubs.

5. *"Table 4. a. Unfortunately, I could not find either the original list of 557 genes nor a list of hub genes in the supplementary material to the article. b. The article did not specify what background was used to calculate GO enrichment. Since the enrichment of a subset from 557 genes is being studied, it makes sense to use these 557 genes as a background."*

Reply: We thank the reviewer for the comment. Following the reviewer 2 and 3's suggestions, we have now changed the entire enrichment analysis (refer to the reply to the Major Comment 8 of the reviewer 2). We have now increased the gene-set size to 1106 (removing genes with CV < 0.02). All the gene names are now provided in S1 Supplementary.

6. *"Figure 9. a. Using only two colors to represent edges makes it hard to understand the figure. Perhaps adding at least one color for edges missing in the LHB would increase the readability of the figure. b. From the above analysis, it is not clear how much of the observed identity between IL and PL, as well as the difference with LHB, is due to biological reality rather than random variation.*

Resampling with replacement of the original data (bootstrap) would help to assess how stable the results are. Some variant of shuffling might provide an indication of the statistical significance of the results found."

Reply: We thank the reviewer for the comment. Using a similar bootstrapping-based approach as the reviewer mentioned, we compared the performance of RFGL and RCFGL in Fig 8 on page 15. We selected 500 genes without replacement from the set of 557 genes, and fitted both RFGL and RCFGL for three values of λ_2. We repeated this procedure ten times. Both the methods detected more IL-PL specific edges compared to LHB-IL or LHB-PL. It indicated that the similarity between the networks of IL and PL was biologically relevant. As mentioned earlier, the gene set enrichment analysis has now been changed and thus, the Figure 9 has also been changed, with a simple color scheme as per the reviewer's suggestion.

Reviewer 2

Major comments:

1. *"In the financial disclosure, "Q.L. was National Institute of General Medical Sciences.. " should be "Q.L. was supported by ..". In the same section, the statement about the role of funders is probably wrong, because as it's written now, it seems that all funders play a role in the research, but from the descriptions of those roles, it seems that they correspond to the role of the authors. Likewise, the statement in the competing interests seems also wrong, the authors should have probably written that they declare that no competing interests exist."*

Reply: We thank the reviewer for the comment. We have now appropriately changed the financial disclosure and the statement of competing interests. The Acknowledgements section of the main manuscript has been made clearer and changed as,

"Q.L. was supported by the National Institute of General Medical Sciences (NIGMS) of the National Institute of Health (NIH) grant R01GM109453. E.B.B. was supported by the NIGMS training grant T32 GM102057 awarded to Pennsylvania State University. L.M.S. and K.K. were supported by the National Institute on Drug Abuse (NIDA) of the NIH under award numbers P30DA044223. L.M.S. was also supported by NIDA under award number P50DA037844 and by the National Institute on Alcohol Abuse and Alcoholism (NIAAA) of the NIH under award number R24AA013162. K.K. was also supported by the National Heart, Lung, and Blood Institute (NHLBI) of the NIH under award number R01HL152735. The content is solely the responsibility of the authors and does not necessarily represent the official views of the National Institutes of Health."

2. *"In the data and code availability statement the authors declare that simulated data is available upon request. That's simply unacceptable from the perspective of this reviewer."*

Reply: We thank the reviewer for the comment. Following the reviewers' suggestions, we have now added fully commented codes used to generate and analyze the datasets of the paper in a folder named "Codes_used_in_paper" to our Github repository, https://github.com/sealx017/RCFGL. The data-generating codes are equipped with the "seeds" we originally used. The codes to generate the figures of the paper along with the extracted results (as rdata files) are also provided.

3. *"pg. 3 In "We use iterative ADMM algorithm .." an article is missing."*

Reply: We thank the reviewer for the comment. We have now added the reference, " Boyd et al. (2011) [2]".

4. *"pg. 8 A theorem, Theorem 1, is given without proof."*

Reply: We thank the reviewer for the comment. We had provided the proof in the supplementary material and we have been mentioned that in the main text of page 8 below the theorem as, "The proof of Theorem 1 can be found in S1 Supplementary."

5. *"pg. 8 In "For example, suppose there three conditions .." the verb "are" is missing."*

Reply: We thank the reviewer for pointing out the error which has now been fixed.

6. *"pg. 9 The specification of the ordering in which RCFGL is applied to each condition is made on the basis of minimizing the distance between every pair of sample covariance matrices derived from each corresponding condition. This distance is calculated here using the Euclidean distance, which is sensitive to large squared terms, which in this context may arise from the instability of these matrices derived from data with many more genes than available samples. This problem has been widely studied, see, for instance, Bryan et al. The comparison of sample covariance matrices using likelihood ratio tests, Biometrika, 1987, and more recent accounts in the field of quantitative*

genetics, such as in Steppan et al. Comparative quantitative genetics: evolution of the G matrix, Trends in Ecology & Evolution, 2002."

Reply: We thank the reviewer for raising a valid point. We do understand that we have provided a fairly simplistic way of determining the ordering of the conditions in the sequential penalty. It is absolutely true that a more sophisticated distance instead of the Euclidean distance between the sample covariance matrices of different conditions could be more robust and the work by Bryan et al. (1987) highlights that. However, we believe that the ordering should be best guided by biological or clinical evidence relevant to the real context. For example, in the modeling of brain networks for Alzheimer's disease in three conditions: normal controls (NC), patients of mild cognitive impairment (MCI), and Alzheimer's patients (AD) using PET scan neuroimaging data, the networks are expected to evolve over time, in the order of disease progression from NC to MCI to AD [18]. Thus, a suitable ordering in this case would be (NC, MCI, AD). We discussed the Euclidean distance-based approach for order detection just as a simplistic and easily interpretable option and it happened to work well in our simulation studies. It is worth mentioning that in our software package, we allow the users to choose their own ordering along with the default option of the Euclidean distance-based approach. We have now added the following paragraph on page 9 of the main manuscript:

"The ordering can be based on biological information available about the degree of similarity across the conditions, for instance, relationship in cell lineages. Alternatively, we can use hierarchical clustering based on the gene expression data, $\mathbf{Y}^{(k)}$, $k = 1, \ldots, K$, and other sophisticated data-driven ways of obtaining a suitable ordering. As an example, here we discuss a simple method based on comparing the sample covariance matrices across the conditions." In the main manuscript, we also studied the sensitivity of our method with respect to the ordering.

7. *"pg 11 The performance is evaluated in terms of TPR, FPR and ROC curves, where true positives (TPs) refer to predicted edges present in the real network and false positives (FPs) refer to predicted edges absent from that network. The decision to cast a TP or FP seems to be made on the basis of the comparison of the cells of the estimated precision matrix with the zero value. However, it is unclear whether this is decided using some tolerance or expecting an exact zero. The authors should clarify. Moreover, in the context where the number of true negatives (TNs), i.e., the number of missing edges, is overwhelmingly larger than the number of TPs the FPR is not a good measure because FPR=FP/(FP+TN) and the very large number of TNs is going make FPR always small. A precision-recall curve, based on the rate of TPs over the total of predictions, i.e., PPV=TP/(TP+FP), is more suitable for such an unbalanced classification problem. In the same page, equation (5) is called the Jaccard Index (see https://en.wikipedia.org/wiki/Jaccard˙index)."*

Reply: We thank the reviewer for the great comment. Following the suggestion, we have now replaced the ROC curves from Figs 2 and 5 with the following precision-recall curves. The tolerance level was kept at 0.01 which we have now mentioned in the main text as well. The main texts on pages 11, 12 and 13 have been changed as:

"We first determined the true and false positives and false negatives in the following way. If the ij-th edge was present in the true network of the k-th condition i.e., $\boldsymbol{\Theta}_{ij}^{(k)} \neq 0$ and was also identified in the estimated network i.e., $|\hat{\boldsymbol{\Theta}}_{i,j}^{(k)}| \geq tol$, it was counted as a true positive (TP). Here, tol was a chosen level of tolerance to define an edge and it was kept at 0.01. Similarly, if the edge was absent in the true network i.e., $\boldsymbol{\Theta}_{ij}^{(k)} = 0$ but was identified in the estimated network, it was counted as a false positive (FP). If the edge was present in the true network but was not identified in the estimated network i.e., $|\hat{\boldsymbol{\Theta}}_{i,j}^{(k)}| < tol$, it was counted as a false negative (FN). Next, the precision ($= \frac{\text{TP}}{\text{TP+FP}}$) and recall ($= \frac{\text{TP}}{\text{TP+FN}}$) were computed to plot the precision-recall curves."

"In scenario (S1), all the methods had nearly identical precision-recall curves for edge detection

Figure 2: **Comparison of edge detection performance for simulations with three conditions.** Top and bottom rows respectively correspond to the precision-recall curves in scenario (S1) and scenario (S2). The x and y axes respectively correspond to recall and precision of the methods for different values of λ_1. Three different values of λ_2 are considered.

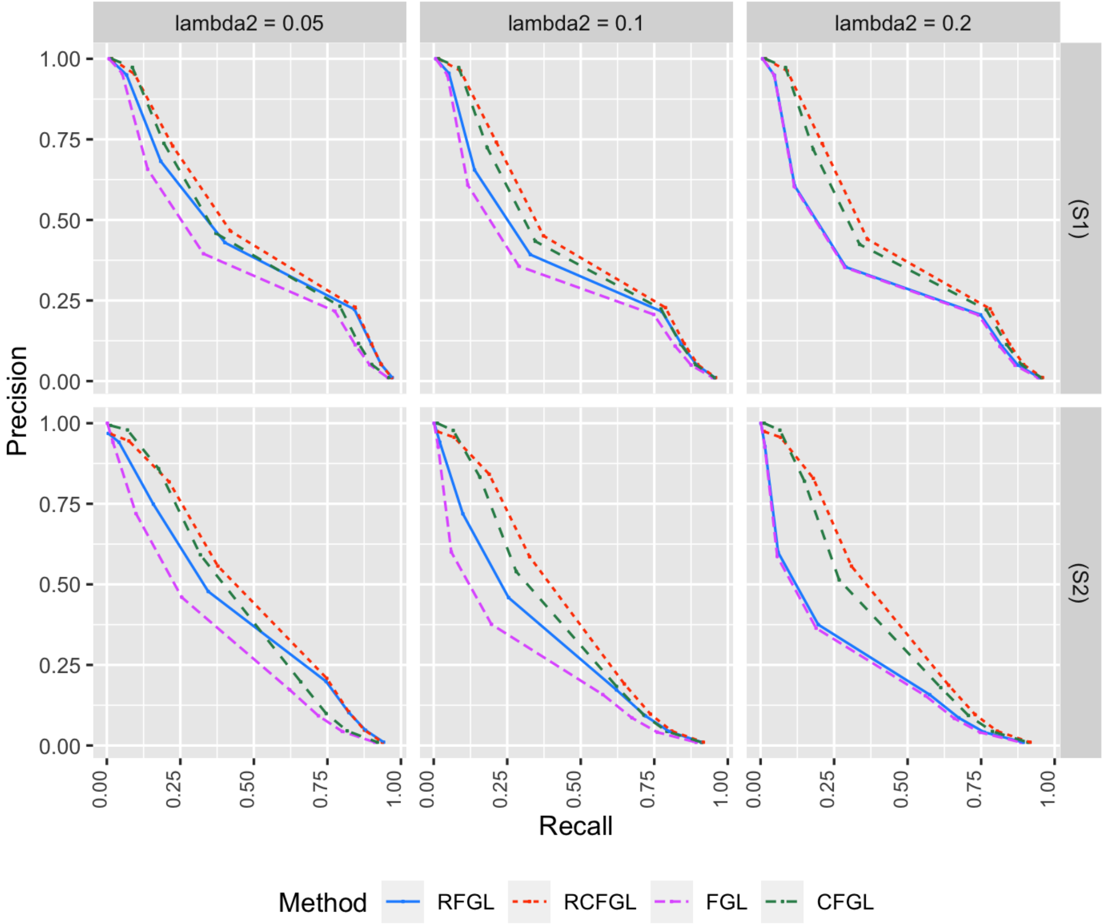

(Figure 2), especially for smaller values of λ_2. However, in scenario (S2) where the assumption of same level of similarity across all the pairs of conditions was violated, CFGL and RCFGL respectively achieved better precision-recall curves than their non-condition adaptive counter-parts FGL and RFGL."

"RCFGL had consistently better precision-recall curves compared to the other methods in all the scenarios (Figure 5). For larger values of λ_2, the precision-recall curve of RFGL was very close

Figure 5: **Comparison of edge detection performance for simulations with three conditions.** Top and bottom rows respectively correspond to the precision-recall curves in scenario (S3) and scenario (S4). The x and y axes respectively correspond to recall and precision of the methods for different values of λ_1. Three different values of λ_2 are considered.

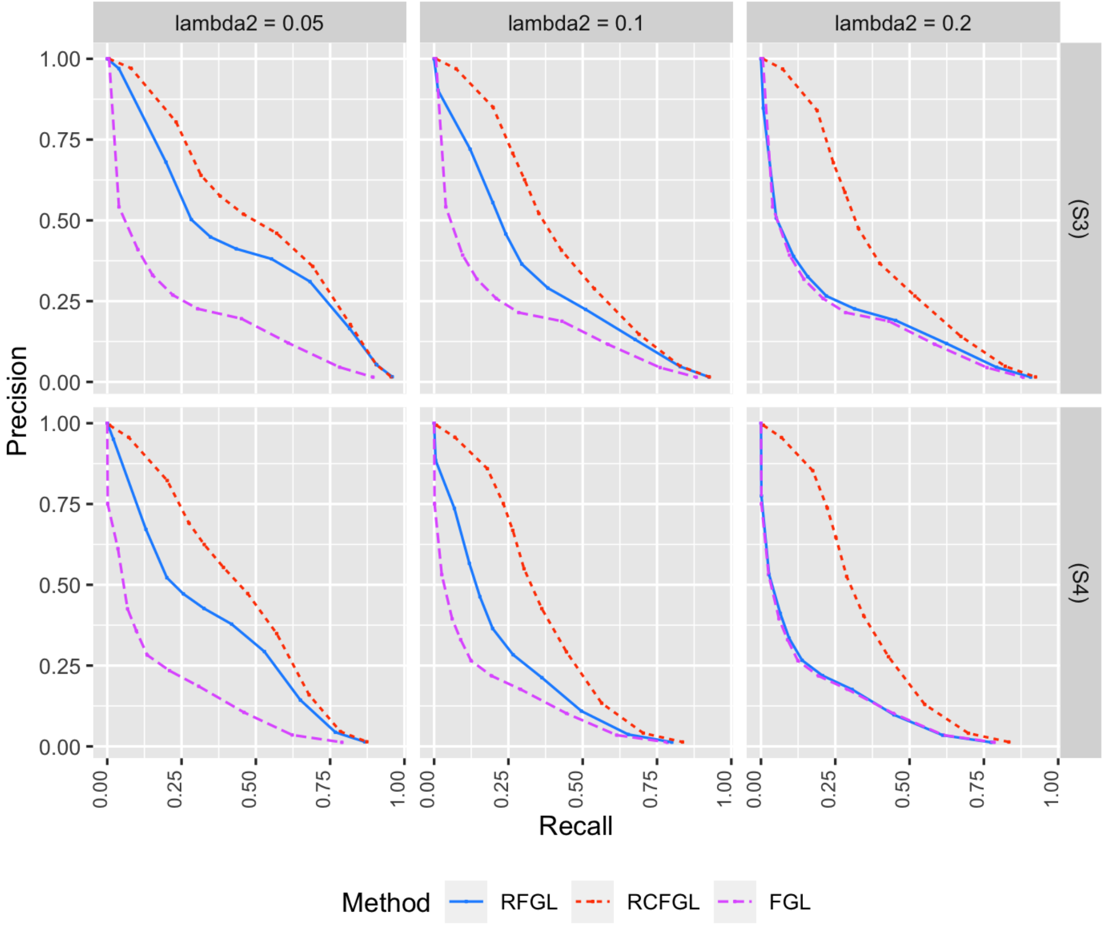

to the curve of FGL."

We have also added the "Jaccard index" in the main text as suggested by the reviewer as: "In mathematical terms, we looked at the following proportion also known as the Jaccard index [11]".

8. *"pg 16 The analysis of real transcriptomics data from brain is somewhat deceptive as there is no rationale behind finding very unspecific pathways among hub genes that are common to the*

conditions. The insight into the brain-specific molecular processes is very poor."

Reply: We thank the reviewer for the valuable comment. We have now entirely changed the gene set enrichment analysis based on the reviewer 2 and 3's comments. We identified the hub-genes from the estimated networks by RCFGL whose degree (i.e., the number of connections) were similar in the prefrontal cortex regions IL and PL but different in LHB. Then, we studied the functional enrichment separately for the two sets of genes: the ones whose degree decreased from IL and PL to LHB and the others whose degree increased from IL and PL to LHB. On page 12, we have added the following paragraph:

" To examine the biological relevance of results from the network analysis by RCFGL, we first identified the hub-genes in every brain region, defined as the genes with more than five connections. Then, we checked which of those hub-genes had similar degree in the medial prefrontal cortex regions IL and PL but different degree in the LHB region. Finally, we studied the functional enrichment separately for the two sets of genes: the ones whose degree decreased from IL and PL to LHB and the others whose degree increased from IL and PL to LHB. Functional enrichment was evaluated using the ShinyGO tool (version 0.76.2; http://bioinformatics.sdstate.edu/go/; [5]) specifying the KEGG Pathway and the Gene Ontology (GO) Biological Process database for simplicity. The pathways with at least three related genes and FDR < 0.05 were reported."

On page 16, we have added the following paragraph:

"Our next goal was to identify biological functions of the hub-genes of the estimated networks by RCFGL using the methodology described in section Measures for evaluating performance. We ran RCFGL on the set of 1106 genes (CV cutoff < 0.02) with $\lambda_1 = 0.01$ and varying values of $\lambda_2 = 0.001, 0.0025, 0.005, 0.01$ and 0.05. The lowest value of AIC was observed for $\lambda_2 = 0.01$ and we interpreted the corresponding network estimates. There were 11 genes that were highly connected in the medial prefrontal cortex regions IL and PL but lost that connectivity in the LHB. These

eleven genes were highly enriched (FDR < 0.01) for “Response to corticosteroid” (GO: 0031960) and similar GO terms. This follows what is known about the differences between these two brain regions. The medial prefrontal cortex has a well-established role as one of the primary sites for stress regulation and as a key site for glucocorticoid actions [12]. In contrast, the LHB is further downstream and receives stress-related signals from the medial prefrontal cortex [9]. Likewise, 57 genes were highly connected in the LHB but lost that connectivity in the medial prefrontal cortex. These genes were enriched for “Intestinal immune network for IgA production” (KEGG Pathway). Microglial cells are the dominant immune-related cell type in brain. Several studies recently have demonstrated the heterogeneity of these cells across brain regions [13, 14], so it is not surprising that the connectivity of genes related to immune response differed across brain regions. Additional enrichment results can be found in Table 4 and 5, respectively listing the top pathways detected using the two sets of hub-genes: the ones whose degree decreased from IL and PL to LHB and the others whose degree increased. Figure 9 shows the estimated sub-networks between these two sets of genes in the three brain regions. We noticed that the sub-networks corresponding to the regions IL and PL were closer to each other than LHB.”

The following sentence has been added to the Discussion section on page 18:

“Finally, we performed enrichment analysis with the hub-genes of the estimated networks by RCFGL whose degree decreased from IL and PL to the LHB region, finding association with stress regulation and glucocorticoid actions.”

Tables 4 and 5 have been added on page 17 and the Figure 9 has been changed as,

Figure 9: **The sub-networks between the hub-genes whose degree changed from IL and PL to LHB.** The top row corresponds to the genes whose degree decreased from IL and PL to LHB, while the bottom row corresponds to the genes whose degree increased.

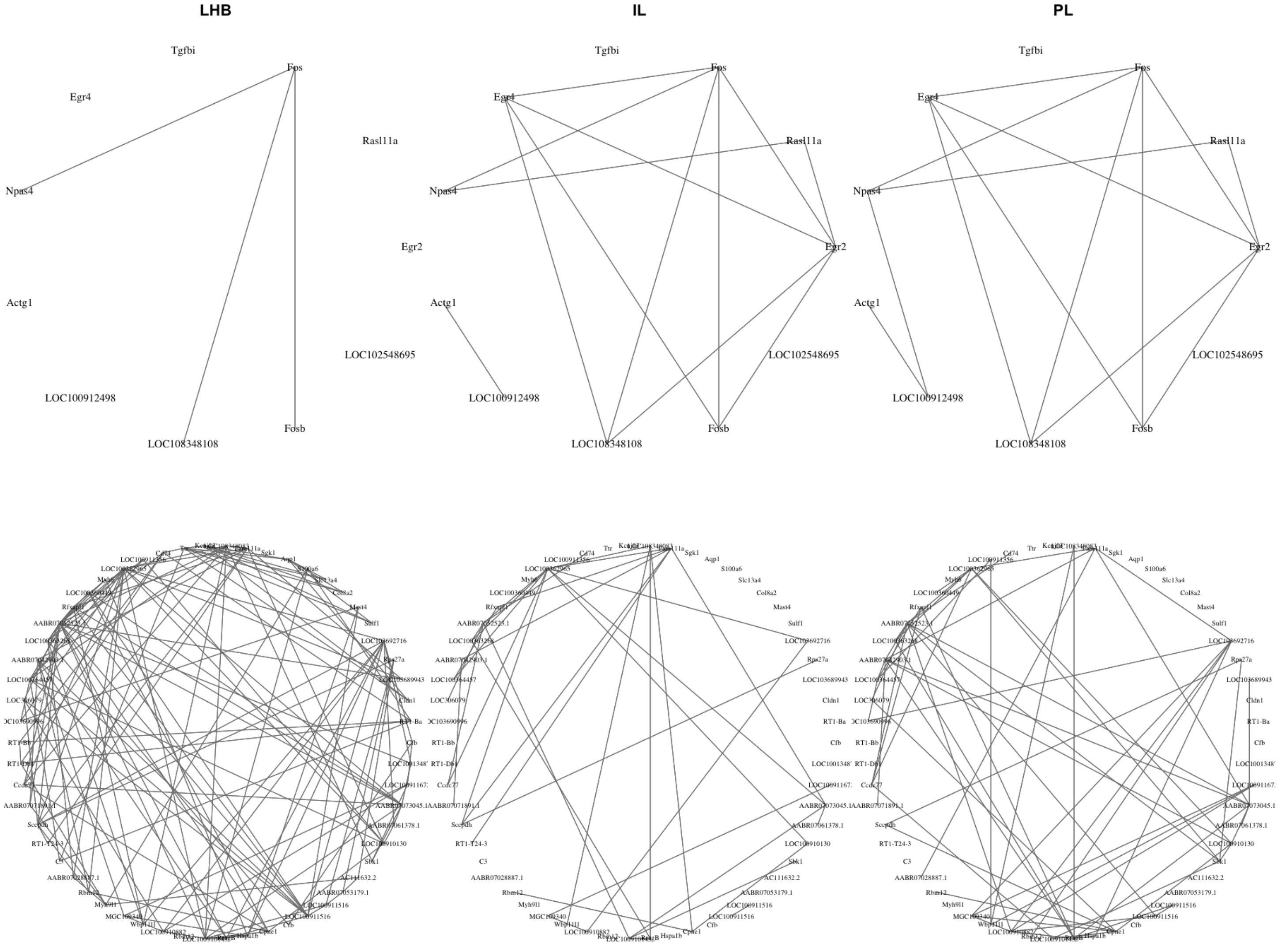

9. *"The software is provided at the GitHub repo https://github.com/sealx017/RCFGL with very poor installation instructions and little guidance on how to use it on a real dataset."*

Reply: We thank the reviewer for the helpful comment. Following all the reviewers' suggestion,

the repo has now been updated with simpler installation instructions and guidance on how to apply the method to a real data set.

Reviewer 3

Major comments:

1. *"1. Performance is evaluated several times using the ROC curve. 1.1. It would be great to see a precision-recall curve instead of a ROC curve. In gene coexpression inference, the number of positive examples are far fewer than the number of negative examples (i.e. the number of gene pairs with true edges between them are far fewer than the number of gene pairs without true edges between them), and precision-recall curves are a better measure of performance than ROC curves in these cases. Furthermore, in biological applications we are generally more interested in the positive edges. Cutoffs are often used in large networks, so specifically the top/most weighted/highest confidence edges are of larger interest. The precision-recall curve is sensitive to changing performance in calling positives correctly without adding a large amount of false positives. 1.2. Edges that are the wrong sign are still counted as true positives in this evaluation. It is unclear whether this is acceptable. There is value in value in being able to identify an edge/relationship that exists, but would struggle to call one method better than another if the method is consistently identifying relationships in the opposite manner in which they actually occur. It would be helpful to show how much the evaluation would change if edges of the wrong sign were not counted as true positives. "*

Reply:. We thank the reviewer for mentioning two great points. To address 1.1, we have now replaced the ROC curves from Figs 2 and 5 with respective precision-recall curves and changed the main text on pages 11, 12 and 13. To view the edits, please refer to the detailed reply to the Major Comment 7 of the reviewer 2.

To answer 1.2, this phenomenon is actually getting evaluated in Figs 3 and 6 of the main manuscript where we compared the sum of squared error (SSE) of the methods. Note that if the true ij-th edge in condition k, $\boldsymbol{\Theta}_{ij}^{(k)}$ is positive and the estimated edge $\hat{\boldsymbol{\Theta}}_{ij}^{(k)}$ is negative, the SSE is going to be increased as the component $(\boldsymbol{\Theta}_{ij}^{(k)} - \hat{\boldsymbol{\Theta}}_{ij}^{(k)})^2$ is going to be large. In all of our simulations, RCFGL achieved the lowest SSE implying it to be best out of the four methods.

2. *"The comparisons across networks are interesting, but certainly expected due to the sequential penalty forcing IL and PL to be more similar to each other than to LHB. Currently, there is no direct evaluation of whether the differing edges between the networks reflect meaningful biological function. The gene enrichment analysis in the manuscript now does not address network-specific genes/edges. Hence, it would be best to move it from the Real data analysis results section to the Supplement and replace it with a new enrichment analysis that focuses on hub genes that were more prevalent in the lateral habenula (LHB) core than the infralimbic (IL) cortex and prelimbic (PL) cortex. As one expects LHB to differ more in function, it would be important to see if the genes that were more "hubby" (higher degree) in LHB compared to IL and PL are enriched for specific functions important for LHB (and vice versa for IL/PL compared to LHB). This could be done by subtracting the average degree of each gene in the IL/PL networks from its degree in the LHB networks and doing enrichment on the genes that meet some threshold for LHB - IL/PL degree."*

Reply: We thank the reviewer for a great suggestion. We have now followed exactly what the reviewer suggested. Please refer to the detailed response to the Major Comment 8 of the reviewer 2 for the changes.

3. *"Coexpression networks are often built using pairwise Pearson/Spearman correlations. These kinds of networks tend to be a measure of which genes are "on and off" together, but do not necessarily imply a causal or even functional relationship between them. The proposed method instead*

calculates a precision matrix, which generally should not contain edges between genes that are conditionally independent. This means, as an example, that one should not expect two genes that are turned on by the same transcription factor to have an edge between them. 3.1. It would be good to include Pearson/Spearman-based coexpression networks as baselines in all the analyses. 3.2. A bit more commentary on the difference between using this method and the commonly-used Pearson/Spearman correlation methods (or situations where it would be more/less advantageous) would be nice to orient people with less math background. This could be added to the introduction where "the co-expression network is constructed based on the dependence structure" is discussed (first paragraph)."

Reply: We thank the reviewer for the great comment. Please refer to our response to the Major Comment 4 of the reviewer 1 in which we addressed a similar point in great detail.

Minor comments:

1. *"There is no code to reproduce the simulated data. Releasing this code improves reproducibility and facilitates later comparisons. Alternatively, these simulated datasets could be added to the Github repository in Data. "*

Reply: We thank the reviewer for the comment. Following the reviewers' suggestions, we have now added fully commented codes used to generate and analyze the datasets of the paper in a folder named "Codes_used_in_paper" to our Github repository, https://github.com/sealx017/RCFGL. The data-generating codes are equipped with the "seeds" we originally used. The codes to generate the figures of the paper along with the extracted results (as rdata files) are also provided.

2. *"2. In methods for the Real data (starting page 10/23): 2.1. The number of rats remaining "after extensive quality control" is given. What quality control measures are used here? The sentence*

being referred to is this: "After extensive 285 quality control, 83 rats remained with RNA-Seq from the lateral habenula (LHB) core, 286 84 rats with data from the infralimbic (IL) cortex, and 82 rats with data from the 287 prelimbic (PL) cortex [54,55]." 2.2. Only 64 rats were used in the final analysis, a supplemental table with sample/run identifiers would be helpful for reproducibility. "

Reply: We thank the reviewer for the comment. Initially, individual libraries were removed if their raw read count was less than 5 million, if more than 2% of reads were removed during trimming, if the average read length after trimming was less than 95 nucleotides, if less than 25% of trimmed reads aligned to the Ensembl transcriptome, if less than 50% of trimmed reads aligned to the reference genome, or if less than 50% of reads that aligned to the genome aligned uniquely. Next, libraries were eliminated from the three brain regions of interest because the genotype information derived from the RNA-Seq data did not match the genotype information from that rat derived from genotype-by-sequencing. Based on the same genotype comparison, two rat labels were swapped within the LHB data. Quality control measures are also documented in [10]. Raw sequencing data can be accessed through GSE173141. We have now added these details and the Rat IDs which were finally used to S1 Supplementary. Added text in the supplementary:

"Raw sequencing data of this paper can be accessed through GSE173141. Initially, individual libraries were removed if their raw read count was less than 5 million, if more than 2% of reads were removed during trimming, if the average read length after trimming was less than 95 nucleotides, if less than 25% of trimmed reads aligned to the Ensembl transcriptome, if less than 50% of trimmed reads aligned to the reference genome, or if less than 50% of reads that aligned to the genome aligned uniquely. Next, libraries were eliminated from the three brain regions of interest because the genotype information derived from the RNA-Seq data did not match the genotype information from that rat derived from genotype-by-sequencing. Based on the same genotype comparison, two rat labels were swapped within the LHB data. Quality control measures are also documented in [10].

In the final analysis, we considered 64 rats whose IDs are listed below for the sake of reproducibility."

`00077E67B5, 00077E8336, 00077EA7E6, 000789FF7D, 00078A0041, 00078A0138, 00078A0189, 00078A01C0, 00078A0224, 00078A02CB, 00078A18A7, 00078A19A7, 00078A19C0, 00078A1F34, 00078A2315, 00078A2463, 00078A261F, 000789FFF0, 000789FFF9, 00078A0139, 00078A01DB, 00078A1707, 00078A1837, 00078A1A16, 000789FF6E, 000789FF94, 000789FFD3, 00078A0058, 00078A0085, 00078A0127, 00078A0166, 00078A01A6, 00078A01D8, 00078A01FE, 00078A021A, 00078A022D, 00078A0255, 00078A02DF, 00078A087B, 00078A09B1, 00078A0AEA, 00078A16D3, 00078A16DF, 00078A1731, 00078A1732, 00078A1772, 00078A179C, 00078A17F7, 00078A1807, 00078A181B, 00078A1863, 00078A186C, 00078A1875, 00078A18CF, 00078A18F7, 00078A192C, 00078A1937, 00078A1942, 00078A194B, 00078A1979, 00078A19B5, 00078A1A2B, 00078A22DF, 00078A22EB, 00078A2595`

References

[1] David Amar, Hershel Safer, and Ron Shamir. Dissection of regulatory networks that are altered in disease via differential co-expression. *PLoS computational biology*, 9(3):e1002955, 2013.

[2] Stephen Boyd, Neal Parikh, and Eric Chu. *Distributed optimization and statistical learning via the alternating direction method of multipliers.* Now Publishers Inc, 2011.

[3] David Roxbee Cox and Nanny Wermuth. *Multivariate dependencies: Models, analysis and interpretation.* Chapman and Hall/CRC, 2014.

[4] Patrick Danaher, Pei Wang, and Daniela M Witten. The joint graphical lasso for inverse covariance estimation across multiple classes. *Journal of the Royal Statistical Society. Series B, Statistical methodology*, 76(2):373, 2014.

[5] Steven Xijin Ge, Dongmin Jung, and Runan Yao. Shinygo: a graphical gene-set enrichment tool for animals and plants. *Bioinformatics*, 36(8):2628–2629, 2020.

[6] Jian Guo, Elizaveta Levina, George Michailidis, and Ji Zhu. Joint estimation of multiple graphical models. *Biometrika*, 98(1):1–15, 2011.

[7] Min Jin Ha, Veerabhadran Baladandayuthapani, and Kim-Anh Do. Dingo: differential network analysis in genomics. *Bioinformatics*, 31(21):3413–3420, 2015.

[8] Min Jin Ha, Junghi Kim, Jessica Galloway-Peña, Kim-Anh Do, and Christine B Peterson. Compositional zero-inflated network estimation for microbiome data. *BMC bioinformatics*, 21(21):1–20, 2020.

[9] Victoria I Hones and Sheri JY Mizumori. Response flexibility: The role of the lateral habenula. *Frontiers in Behavioral Neuroscience*, 2022.

[10] Daniel Munro, Tengfei Wang, Apurva S Chitre, Oksana Polesskaya, Nava Ehsan, Jianjun Gao, Alexander Gusev, Leah C Solberg Woods, Laura M Saba, Hao Chen, et al. The regulatory landscape of multiple brain regions in outbred heterogeneous stock rats. *bioRxiv*, 2022.

[11] Allan H Murphy. The finley affair: A signal event in the history of forecast verification. *Weather and forecasting*, 11(1):3–20, 1996.

[12] Brent Myers, Jessica M McKlveen, and James P Herman. Glucocorticoid actions on synapses, circuits, and behavior: implications for the energetics of stress. *Frontiers in neuroendocrinology*, 35(2):180–196, 2014.

[13] Vassilis Stratoulias, Jose Luis Venero, Marie-Ève Tremblay, and Bertrand Joseph. Microglial

subtypes: diversity within the microglial community. *The EMBO journal*, 38(17):e101997, 2019.

[14] Yun-Long Tan, Yi Yuan, and Li Tian. Microglial regional heterogeneity and its role in the brain. *Molecular psychiatry*, 25(2):351–367, 2020.

[15] Bruno M Tesson, Rainer Breitling, and Ritsert C Jansen. Diffcoex: a simple and sensitive method to find differentially coexpressed gene modules. *BMC bioinformatics*, 11(1):1–9, 2010.

[16] Robert Tibshirani. The lasso method for variable selection in the cox model. *Statistics in medicine*, 16(4):385–395, 1997.

[17] Robert Tibshirani, Michael Saunders, Saharon Rosset, Ji Zhu, and Keith Knight. Sparsity and smoothness via the fused lasso. *Journal of the Royal Statistical Society: Series B (Statistical Methodology)*, 67(1):91–108, 2005.

[18] Sen Yang, Zhaosong Lu, Xiaotong Shen, Peter Wonka, and Jieping Ye. Fused multiple graphical lasso. *SIAM Journal on Optimization*, 25(2):916–943, 2015.
