# Supplementary material for: RCFGL: Rapid Condition adaptive Fused Graphical Lasso and application to modeling brain region co-expression networks
Source: PLoS Comput Biol. 2023 Jan 6;19(1):e1010758. doi: 10.1371/journal.pcbi.1010758 (PMC9821764; doi:10.1371/journal.pcbi.1010758)
Supplement: Attachment — Submitted filename: comments.docx [file pcbi.1010758.s007.docx]

**Comments to the Authors:**

This article is a continuation of work published by the same group in the journal PLOS Computational Biology in 2018. The presented paper focuses on the analysis of differential gene co-expression networks. This task is a fundamental and challenging problem for identifying gene interactions and regulatory relationships under different conditions. Analysis of gene expression data is widely used in transcriptomic studies to understand the functions of molecules in the cell and the interactions between them. Differential co-expression analysis allows studying the diversity of diseases and phenotypes by identifying gene modules with distinct co-expression patterns under multiple conditions. A co-expression network analysis allows researchers to identify groups of highly correlated genes that are associated with biological processes or pathways and displays the correlation between genes. Understanding the differential co-expression of genes underlying molecular mechanisms can help uncover pathological mechanisms and underlying regulatory dynamics that cannot be detected by differential expression analysis of individual genes alone. It also helps to understand disease progression, separate diseases, and identify potential biomarkers responsible for phenotypic changes or severe disease.

Currently, the volume of transcriptome data is steadily increasing and new fast algorithms for analysis are required. This problem has been successfully solved by the authors.

Despite the overall clarity and elegance of the article, I had a few critical comments and suggestions for the authors of this paper:

Major comments:

1) In my opinion, this article together with the wonderfully designed repository may be of interest to a wide range of biologists. I was fully satisfied with the detailed mathematical description of the method, but to broaden the scope of readers I would suggest adding didactic figures and schemes explaining the principle of the method. I would also expand a little for non-experienced readers on the concept and necessity of L1 and L2 regularizations.
2) Despite the publicly available and well-documented implementation of the method, I could not find any code for the figures in this article. Publishing the code is not only necessary as part of open science and journal policy, but will also greatly assist followers in discovering the potential of your method.
3) One of the main transcriptomic methods at the moment is single-cell sequencing. The data obtained by this method do not meet the assumptions of normality and often contain a significant amount of missing data. Despite the fact that such data were used in this article, I did not see any discussion of how sensitive the proposed method is to the assumption of normality and how to perform (and if at all) the imputation of missing measurements.
4) Despite a good description of methods close to RCFGL, the authors left out a description and comparison with other methods of establishing differential co-regulatory networks, such as DiffCoEx, WGCNA, DICER, DINGO, etc.
5) One of the main claims of this article was the acceleration of existing methods. Unfortunately, these results were poorly expressed in the abstract, where I would add more concrete numbers on the increase in the speed of network construction with the proposed algorithm. I would also like to see a comparison of different algorithms, not only on simulated data but also on real data.

Minor comments:

1) After reading the paper, it remains unclear to me what happens if two genes in one group are linked positively and the other are linked negatively. Will such an edge be preserved as common for the two groups, or as different?
2) Page 10, The legend to figure 1 could be more detailed.
3) Page 15, lines 444-446. Assessing the significance of observed differences would greatly strengthen the claim.
4) Page 16, lines 465-467. Based on what data/reasoning was the number five chosen as the treshold for categorizing the gene as a hub?
5) Table 4.
   a. Unfortunately, I could not find either the original list of 557 genes nor a list of hub genes in the supplementary material to the article.
   b. The article did not specify what background was used to calculate GO enrichment. Since the enrichment of a subset from 557 genes is being studied, it makes sense to use these 557 genes as a background.
6) Figure 9.
   a. Using only two colors to represent edges makes it hard to understand the figure. Perhaps adding at least one color for edges missing in the LHB would increase the readability of the figure.
   b. From the above analysis, it is not clear how much of the observed identity between IL and PL, as well as the difference with LHB, is due to biological reality rather than random variation. Resampling with replacement of the original data (bootstrap) would help to assess how stable the results are. Some variant of shuffling might provide an indication of the statistical significance of the results found.
